# Chagas disease-specific antigens: characterization of epitopes in CRA/FRA by synthetic peptide mapping and evaluation by ELISA-peptide assay

**DOI:** 10.1186/1471-2334-13-568

**Published:** 2013-12-03

**Authors:** Carolina G Bottino, Luciano P Gomes, José B Pereira, José R Coura, David William Provance, Salvatore G De-Simone

**Affiliations:** 1 Centro de Desenvolvimento Tecnológico em Saúde (CDTS)/Instituto Nacional de Ciência e Tecnologia de Inovação em Doenças Negligenciadas (INCT-IDN), Fundação Oswaldo Cruz, Rio de Janeiro, RJ, Brazil; 2 Laboratório de Bioquímica de Proteínas e Peptídeos, Instituto Oswaldo Cruz, Rio de Janeiro, RJ, Brazil; 3 Laboratório de Doenças Parasitárias, Instituto Oswaldo Cruz, FIOCRUZ, Rio de Janeiro, RJ, Brazil; 4 Departamento de Biologia Celular e Molecular, Instituto de Biologia, Universidade Federal Fluminense, Niterói, RJ, Brazil

**Keywords:** Chagas disease, *Trypanosoma cruzi*, Cytoplasmic repetitive antigen, Flagellar repetitive antigen, Epitopes, Spot-synthesis, Peptide-ELISA

## Abstract

**Background:**

The identification of epitopes in proteins recognized by medically relevant antibodies is useful for the development of peptide-based diagnostics and vaccines. In this study, epitopes in the cytoplasmic repetitive antigen (CRA) and flagellar repetitive antigen (FRA) proteins from *Trypanosoma cruzi* were identified using synthetic peptide techniques and pooled sera from Chagasic patients. The epitopes were further assayed with an ELISA assay based on synthetic peptides.

**Methods:**

Twenty-two overlapping synthetic peptides representing the coding sequence of the *T. cruzi* CRA and FRA proteins were assessed by a Spot-synthesis array analysis using sera donated by patients with Chagas disease. Shorter peptides were selected that represented the determined epitopes and synthesized by solid phase synthesis to evaluate the patterns of cross-reactivities and discrimination through an ELISA-diagnostic assay.

**Results:**

The peptide Spot-synthesis array successfully identified two IgG antigenic determinants in the CRA protein and four in FRA. Bioinformatics suggested that the CRA antigens were unique to *T. cruzi* while the FRA antigen showed similarity with sequences present within various proteins from *Leishmania sp.* Subsequently, shorter peptides representing the CRA-1, CRA-2 and FRA-1 epitopes were synthesized by solid phase synthesis and assayed by an ELISA-diagnostic assay. The CRA antigens gave a high discrimination between Chagasic, Leishmaniasis and *T. cruzi-*uninfected serum. A sensitivity and specificity of 100% was calculated for CRA. While the FRA antigen showed a slightly lower sensitivity (91.6%), its specificity was only 60%.

**Conclusions:**

The epitopes recognized by human anti-*T. cruzi* antibodies have been precisely located in two biomarkers of *T. cruzi*, CRA and FRA. The results from screening a panel of patient sera through an ELISA assay based on peptides representing these epitopes strongly suggest that the sequences from CRA would be useful for the development of diagnostic reagents that could improve upon the sensitivity and specificity of currently available diagnostic tests. Overall, the results provide further evidence of the usefulness of identifying specific linear B-cell epitopes for improving diagnostic tools.

## Background

Chagas disease is caused by the protozoan *Trypanosoma cruzi*, which belongs to the Trypanosamatidae family [1]. Infection with *T. cruzi* is endemic across 18 countries of Latin America with an estimated 16 to 18 million cases and up to 120 million additional people are at risk [2]. During the chronic phase of the disease, diagnosis of an infection relies on serological assays since there is a major decline in the number of parasites circulating in patients’ blood [3,4]. The most common techniques used are ELISA, indirect hemagglutination (IH), indirect immunofluorescence (IIF), western blot and immunochromatography [4,5]. While these methods are usually simple to perform and have a low cost, they also can demonstrate low sensitivity and/or specificity, or even cross-reactions with other pathogens, especially *Leishmania spp*. Cross-reactivity is a major concern when whole or semi-purified extracts of *T. cruzi* epimastigotes are used as antigens in serological tests [5].

The antigenic determinants used as binding targets for antibodies can be divided into two categories: linear or nonlinear. Linear epitopes consist of amino acid residues that are adjacent to each other in the primary sequence while nonlinear epitopes consist of amino acid residues that are separated in the primary structure but are brought into proximity when the protein is in its native form. At present, there is no simple way to identify nonlinear epitopes in the absence of three-dimensional structural information displaying antibody-antigen complexes, normally with monoclonal antibodies (mAb). However, the identity of linear epitopes can be predicted by computer programs that calculate various parameters that have been discovered to be correlate with the antigenic nature of previously studied antigens (e.g., hydrophilicity, flexibility and surface probability) [6]. The methods postulate that (a) antibodies bind to linear epitopes by reacting with segments of 4–8 consecutive amino acid residues and (b) these epitopes are situated on the surface of molecules, which tend to be hydrophilic. However, computational techniques are not yet sufficiently sophisticated to achieve the accuracy of experimental techniques. Other methods for identifying antibody binding sites involve: (a) proteolysis of the antigen, (b) recombinant techniques, (c) phage display, (d) mass spectrometry and (e) the use of synthetic peptides. Fragments of antigens derived from trypsin [7] or papain [8] digestion have been used to determine antibody binding targets. Numerous attempts utilizing cyanogen bromide cleavage products have been published [9,10]. The use of recombinant DNA techniques for epitope mapping has been reported [11], including the application of phage display technique to map epitopes in various proteins [12,13]. Another approach applies modern mass spectrometry techniques to locate epitopes [14].

A more robust approach has been the use of libraries of synthetic peptides. Geysen et al. [15] published a method for identifying linear epitopes by using overlapping synthetic peptides from known sequences. Given the recent progress in methods for the simultaneous synthesis of a large number of peptides, it is now practical to create arrays of the corresponding peptides to all possible contiguous segments of a protein of interest. The peptides are designed with sufficient overlapping regions to contain the minimal binding sequence. Linear epitopes are then defined by identifying the peptides that are most strongly associated with antibodies developed against the full-sized antigen. This methodology has been used successfully in numerous cases [16-19].

For Chagas disease, various antigens have been used to improve the diagnosis of Chagas disease. Among them, repetitive proteins (RP) represent very promising targets, as they are usually highly antigenic [4,20,21]. Two well characterized RP’s are the cytoplasmic repetitive antigen (CRA) and the flagellar repetitive antigen (FRA). CRA is a 225 kDa protein composed of a 14-amino acid repeat that is distributed in the cytoplasm of the replicating epimastigote and amastigote forms. FRA is a 300 kDa protein composed of a 68-aminoacid repeat that is located at the flagellum in all stages of the *T. cruzi* life cycle [4,20,22]. These proteins are highly antigenic and induce the production of several classes of antibody in humans [22-24]. They were first used in combination for diagnostic test in 1992, showing good specificity and sensitivity [25]. Other studies also indicated that, used together, CRA and FRA could successfully serve as antigens in diagnostic tests for Chagas disease [26-28].

Despite the widespread use of CRA and FRA in a chimeric form (CF-chimera) as a clinical marker of Chagas disease [25,28,29], little is known about the actual antigenic determinants in CRA/FRA that are involved in and responsible for antibody recognition [21,22,28,30]. Information concerning the composition and the locations of the major epitopes CRA and FRA should provide insight into the antigenic properties of the protein. Furthermore, specific details on the interactions between CRA and FRA with particular antibodies could advance the development of novel antigens possessing characteristics desirable for use in diagnostic assays. Such improvements include higher sensitivity, greater specificity and the elimination of cross-reactions. To achieve the aim of mapping specific epitopes in both CRA and FRA proteins, we used the parallel overlapping synthetic peptide method of Spot-synthesis to create a peptide library of CRA and FRA. This library was probed with a panel of sera from patients with Chagas disease that originated from different endemic areas of Brazil. Based on their reactivity, epitopes were identified and their sequences used to develop an ELISA-peptide assay to be used on the serological diagnosis of Chagas disease.

## Methods

### Materials

Super Signal® West Pico and chemiluninescent substrate were from Pierce Biotechnology (Rockford, IL, USA). Amino-PEG_500_-UC540 cellulose membranes were obtained from Intavis AG Bioanalytical Instruments (Germany). Peroxidase labeled rabbit anti-human immunoglobulin and ABTS peroxidase substrate were from KPL (Gaithersburg, MD, USA). Sephadex® G-25 was from Boehringer-Mannheeim (Ingelheim, Germany) and neutravidin-coated 96-well plates from Thermo Fischer Scientific (Waltham, MA, USA). Bovine serum albumin, 3,3′,5,5′ tetramethylbenzidine (TMB), pyperidine, acetonitrile and trifluoracetic acid and Tween 20 were obtained from Sigma-Aldrich Corp. (St. Louis, MO, USA) and Centricon 10 filters from Amicon (CA, U.S.A.). CDP-Star® Substrate was from Applied Biosystems (Grand Island, NY, USA). The biotin-X-NHS labeling Kit, horseradish peroxidase (HRP) conjugated sheep anti-human IgG, amino acids for peptide synthesis, sequence reagents and all other chemical reagents were from Calbiochem-Merck (Darmstadt, Germany).

### Human sera

Sera from patients chronically infected with *T. cruzi* (n = 31) and *L. brasiliensis* (n = 14) were obtained from the Parasitic Diseases Laboratory (IOC/FIOCRUZ) and Gonçalo Moniz Research Center/FIOCRUZ-Bahia, respectively. The Chagasic patients were from Vergim da Lapa (State of Minas Gerais) and with Leishmaniasis from the state of Bahia. Their diagnosis was confirmed both clinically (through the clinical signs and symptoms) and serologically (IFI and ELISA) before inclusion in the study. Sera from unaffected donors (negative control, n = 11) was obtained from the blood bank of Rio de Janeiro (HEMORIO).

### Synthesis of the cellulose-membrane-bound peptide array

The entire sequences of both CRA and FRA were covered by the synthesis of 10 and 12 peptides, respectively, each with 14 amino acids and offset from the previous by nine amino acids. The peptides were automatically prepared onto Amino-PEG_500_-UC540 cellulose membranes according to standard SPOT synthesis protocols [31] using an Auto-Spot Robot ASP-222 (Intavis Bioanalytical Instruments AG, Köln, Germany). Coupling reactions were followed by acetylation with acetic anhydride (4%, v/v) in N, N-dimethyformamide to render peptides unreactive during the subsequent steps. After acetylation, Fmoc protective groups were removed by the addition of piperidine to render nascent peptides reactive. Amino acids were added sequentially by this same process of coupling, blocking and deprotection until the desired peptide was generated. After addition of the last amino acid in the peptide, the amino acid side chains were deprotected using a solution of dichloromethane–trifluoracetic acid–triisobutylsilane (1:1:0.05, v/v/v) and washed with methanol. Membranes containing the library of synthetic peptides were either probed immediately or stored at −20°C until needed. Positive and negative controls were included in each membrane. The positive control consisted of a spot with the peptide, IHLVNNESSEVIVHK, from *Clostridium tetani* precursor and the negative control was a spot without peptide.

### Screening of SPOT membranes

SPOT membranes were washed with TBS (50 mM Tris-buffer saline, pH 7.0) and then blocked with TBS-CT (Tris-buffer saline, 3% casein, 0.1% Tween 20, pH 7.0) at room temperature under agitation or overnight at 4°C. After extensive washing with TBS-T (Tris-buffer saline, 0.1% Tween 20, pH 7.0), membranes containing the peptide libraries were incubated for 2 h with sera pool from six different Chagasic patients (1:250) in TBS-CT and then washed again with TBS-T. Next, membranes were incubated with alkaline phosphatase labeled rabbit anti-human IgG (1:5000 in TBS-CT) for 1 h, washed with TBS-T and a final wash in CBS (50 mM citrate-buffer saline, pH 7.0). Chemiluminenscente CDP-Star® Substrate (0.25 mM) with Nitro-Block-II™ Enhancer (Applied Biosystems, USA) was added to complete the reaction.

### Scanning and measurement of spot signal intensities

The measurement of spot signal intensities were performed as described previously [32]. Briefly, the chemiluminescent signals were measured on a MF-ChemiBis 3.2 (DNR Bio-Imaging Systems, Israel) and a digital image file generated with a resolution of 5 MP. Signal intensities were quantified with TotalLab Software (Nonlinear Dynamics, USA) using algorithms that compared the intensity between background, spot area and negative control to define the empirical probability that the spot signal intensity was distinct from background signals. The spot with the strongest reactivity on the membrane was defined as an intensity of 100% and all other intensities were expressed as values relative to this intensity.

### Peptide synthesis

Peptides consisting of the sequences of the identified and selected epitopes from the screening of the CRA and FRA peptide libraries were synthesized by solid phase using the F-moc strategy and an automatic synthesizer (PSS-8-Shimadzu, Japan). Epitope sequences (CRA-1 = AAKQKAAEAAAKQKAAEC; CRA-2 = AAKQRAAEAAAKQR AAEC) were each linked in tandem with the insertion of three Ala and were extended by the addition of extra alanine residues on the N- and Cys to the C- terminus to improve presentation of antigens to antibodies and bind to neutravidin during diagnostic tests after biotin labeling. All fluorenylmethoxy carbonyl amino acid derivatives were used to protect the a-amino group and purchased from Novabiochem (San Diego, CA, USA). To protect the side chain, triphenylmethyl was used for Gln and Asn, t-butyl for Thr, Glu, Ser and Asp, t-butyloxycarbonyl for Lys, and 2,2,5,7,8-pentamethylchroman-6-sulphonyl for Arg.

Cleavage of the peptide from the resin was achieved by treatment with a mixture of trifluoroacetic acid: thioanisole: ethane dithiol: water (volume ratios 80:5:2.5:5) at room temperature for 12 h. After filtration and washing of the resin with trifluoroacetic acid, a gentle stream of nitrogen was used to remove excess trifluoroacetic acid. The crude peptide was precipitate at −20°C with diethyl ether and then centrifuged at 3000 g for 10 min at 10°C. The synthetic peptide was purified by high performance liquid chromatography (HPLC) using a reverse phase column (Vydac C18). The purified peptides were characterized by amino acid analysis and electronspray ionization MS at the National Institute of Quality Control on Health (INCQS) of FIOCRUZ, Rio de Janeiro, Brazil.

### Peptide biotinylation

Peptides (CRA-1 = AAKQKAAEAAAKQKAAEC; CRA-2 = AAKQRAAEAAAKQRA AEC and FRA-4 = ADRAFLDQKPERVPC) were labeled with biotin using the Biotin-X-NHS Kit, which allowed attachment to solid supports through association with immobilized neutravidin, as previously described [33]. Briefly, a solution of biotin-NHS (40 μg/μL) prepared in 0.1 M DMF was added to each peptide (10 μg/μL) dissolved in Na_2_HCO_3_/Na_2_CO_3_ to give a volume ratio of 1:4 in the final solution. The peptides were incubated at 25°C for 2 h with stirring followed by purification on a Sephadex® G-25 column using a flow rate of 1 ml/min. Collected biotin-labeled peptide was lyophilized and stored at 4°C until use. The labeling efficiency was determined according to the protocol of the manufacturer.

### Determination of the peptide concentration

The concentration of the peptides was estimated using the ExPASy ProtParam tool at 205 nm using a molar extinction coefficient (http://www.basic.northwestern.edu/biotools/proteincalc.html) previously defined. Alternatively the concentration was determined by an automatic amino acid analyzer (Shimadzu, Kyoto, Japan).

### Molecular modeling

3D structure data was not available for either protein. To obtain a theoretical model, the primary sequences of CRA (access code Q26907) and FRA (access code Q26921) of *T. cruzi* were accessed nine times before submission to I-TASSER server (http://zhanglab.ccmb.med.umich.edu/I-TASSER/) [34,35], which automatically built a predicted model. Visualization was performed using *Visual Molecular Dynamic* (VMD) 1.9 [36]. Models show the correct topology based on TM-score values greater than 0.50, which is a reasonable quality c-score and within accepted limits, and demonstrated greater than 90% confidence for the prediction of the FRA model (c-score = −1,57) and slightly lower for the CRA model (c-score = −1,49).

### ELISA

ELISA-peptide assays were performed as described previously [37] using neutravidin coated plates. Briefly, neutravidin-coated 96-well plates were incubated with 2 mg/mL of each biotin-labeled peptide (100 μL/well) for 2 h at room temperature. After three washes with a wash solution (TBS pH 7.2, containing 0.05% of Tween 20 and 0.1% skimmed milk), diluted human sera (1:50) was added and incubated for 30 minutes at room temperature. Following three washes in wash solution, the sheep anti-human IgG antibody coupled with horseradish peroxidase (HRP) was added at a dilution of 1:14,000 in wash solution and incubated for 30 minutes at room temperature. After three washes, 100 μL of ABTS peroxidase substrate was added to each well and the optical density (OD) was measured at 405 nm following a 30 minute incubation.

### Analysis of the experimental data

The results of ELISA were analyzed through the Receiving Operator Characteristic (ROC) curves, which are equivalent to Wilcoxon statistics [38,39]. Cut-off values were determined using the ROC curve analysis [40]. All statistical analyses were performed with SigmaPlot 10.0 software (Systat Software Inc., Chicago, USA).

## Results

### Linear epitope mapping

Linear epitopes often contain between two and six amino acids that are critical for recognition by an antibody [41,42]. Together with advances in automated peptide synthesis make, it is feasible to generate peptide arrays that span the entire amino acid sequence of a protein. Considering the potential for the *T. cruzi* RP’s CRA and FRA to act as antigens for diagnosing infections, two libraries of peptides were designed to cover the amino acid sequence for each. By screening each library with a pool of serum from Chagas patients (n = 6), each of the antigenic epitopes could be identified for further analysis.

**Figure 1:**
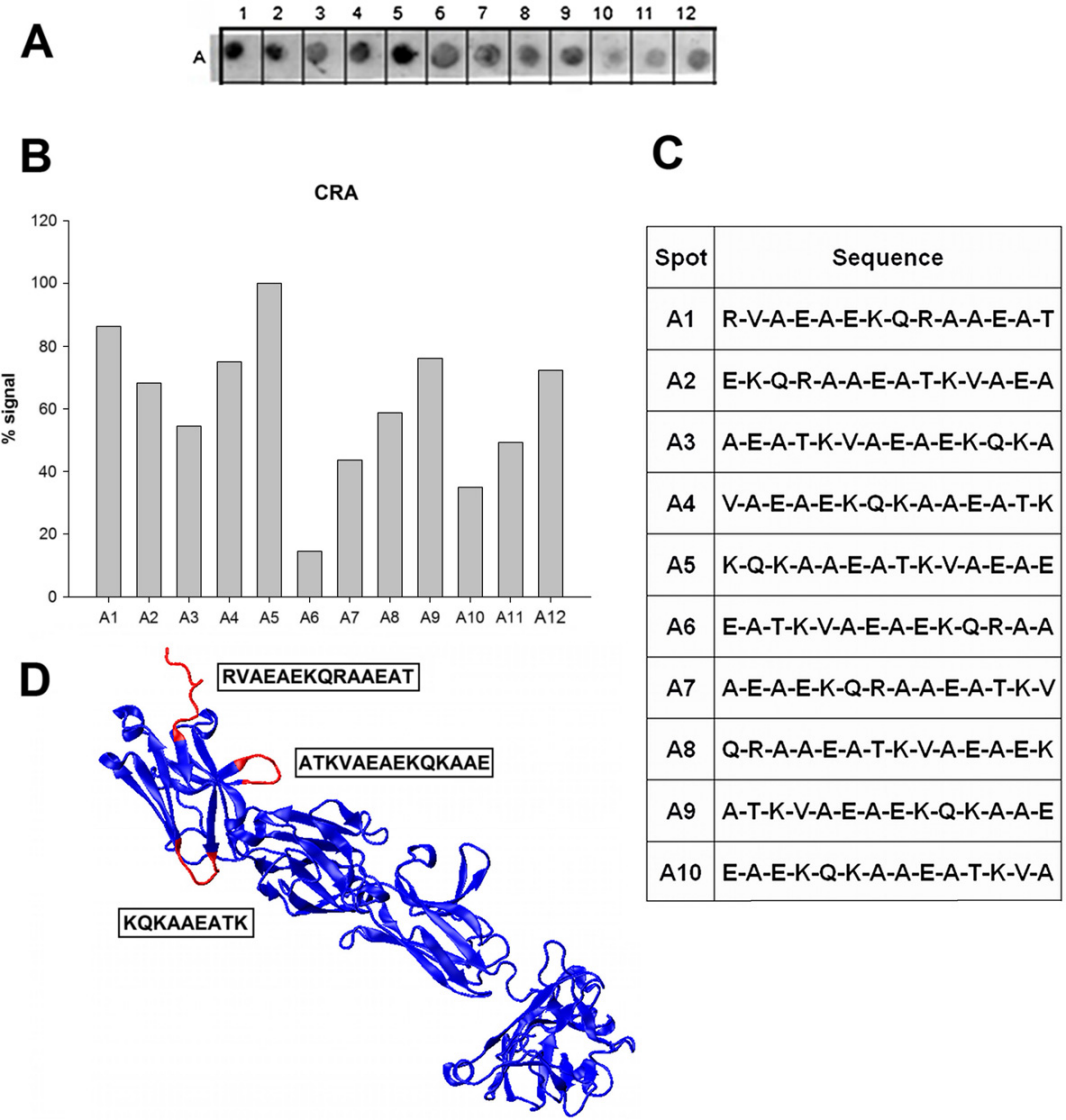
**Linear epitope mapping of CRA with human patient sera.** Peptides consisting of 14 amino acid residues with an overlap of 9 residues between peptides were tested for reactivity against a pool of sera from six Chagasic patients. **(A)** Image of a representative membrane revealed by chemiluminescence after immunoblotting. **(B)** Measured signal intensity of the spots from membrane in Panel A. **(C)** Peptide sequences synthesized and the position of their spots. **(D)** Molecular modeling of CRA protein predicted by I-Tasser server. The visualization of the proteins was executed as a “new cartoon” representation with the quality set at the maximum value (50). The red segments are overlapping epitopes with the greatest signals contrasted against the rest of repeated structure in blue color.

Ten peptides were sufficient to represent the amino acid sequence of CRA and were synthesized in place onto membranes. This peptide array was screened initially with sera pooled from six patients with Chagas disease. The recognition and binding by anti-CRA antibodies was detected by chemiluminescence. A representative image is shown in Figure 1A demonstrating that each peptide was reactive to some extent. A plot of the quantified signal intensities clearly identified the epitopes with the greatest reactivity (Figure 1B). The profile of antigenicity was not dependent on which sera were included in the pools as different pools of sera resulted in the same profiles suggesting that the reactivity was independent of the possible variability in the relative abundance of antibody clones that were reactive with each particular epitope in the proteins (data not shown).

With the sequences that constitute each peptide (Figure 1C), the epitopes were characterized by a theoretical model of their structure. The three major antigenic regions identified had very similar sequences with the only difference between them being the amino acid in the third position; a lysine residue in CRA-1 (7**KQKAAE**15**)** and an arginine in CRA-2 & CRA-5 **(KQRAAE)**. As a consequence, all of the 9 amino acid sequences were modeled with very similar structures composed of a random coil (CRA-1) or as a turn (CRA-2 &-5) as predicted by the I-TASSER server. Their locations, according to the predicted model, also supported their potential to interact with antibodies in solution (in red, Figure 1D). The first peptide, CRA-1, was located at the C-terminus and appeared to be exposed to the solvent. The second and third peptides, CRA-2 and CRA-5, appeared to form a loop on the surface of the protein. The peptide A5 was situated at the end of ß-sheets, which could explain its strong signal.

**Figure 2:**
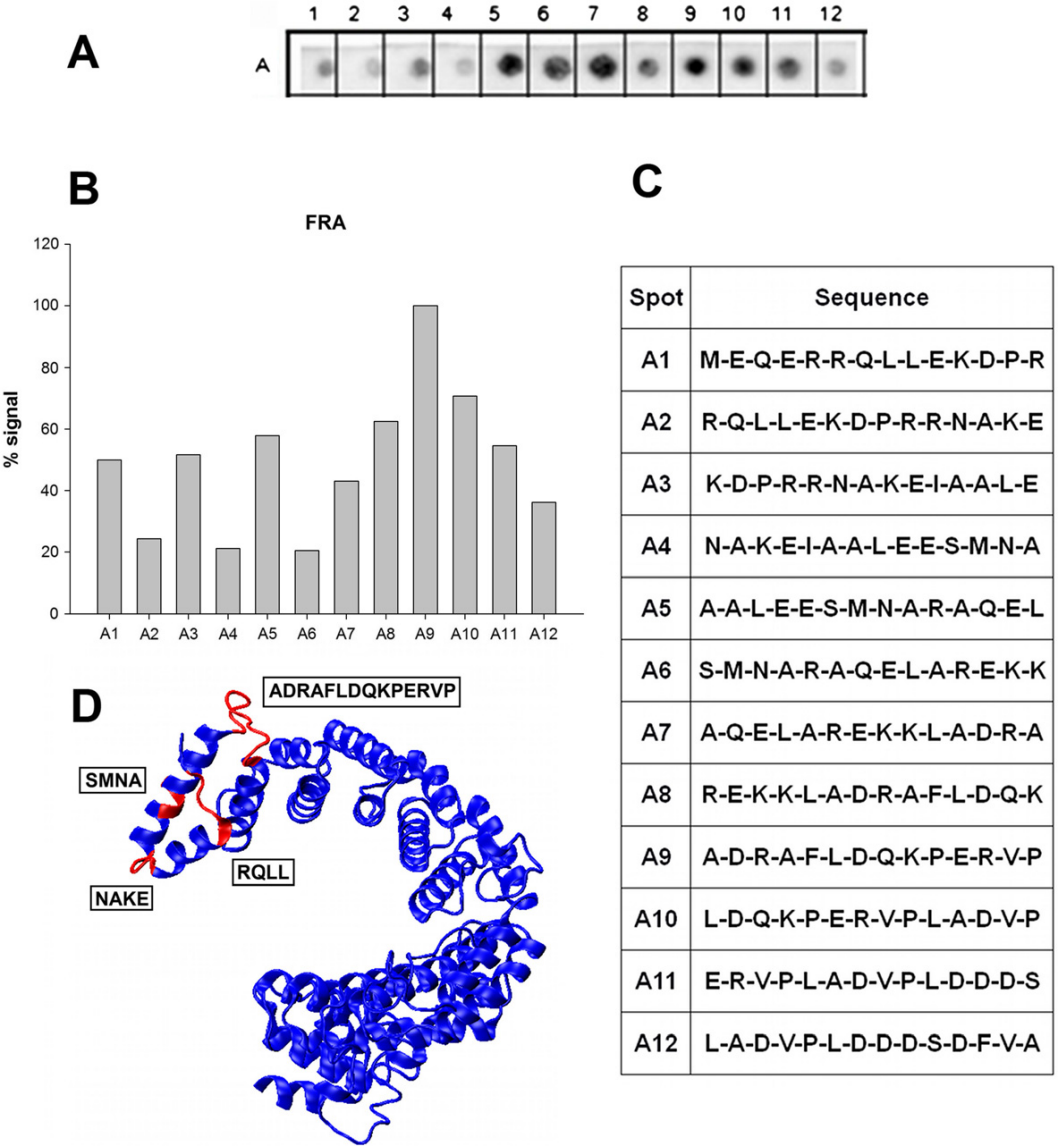
**Linear epitope mapping of FRA with human patient sera.** Peptides 14 amino acid residues long with an overlap of 9 residues between peptides were tested for reactivity against a pool of sera from six Chagasic patients. **(A)** Image of a representative membrane revealed by chemiluminescence after immunoblotting. **(B)** Measured signal intensity of the spots produced from the image in Panel A. **(C)** Sequences of the peptides synthesized and their positions on the membrane. **(D)** Molecular model of the FRA protein as predicted by I-Tasser server. Visualization of proteins was generated as a “new cartoon” representation with the quality of visualization was set at the maximum value of 50. The red segments display the overlapping epitopes with highest signals amongst the rest of the repeated structure in blue.

Equivalent experiments were performed to identify linear B-cell epitopes in FRA using twelve peptides. In Figure 2A, a representative image of a membrane containing the peptide array demonstrated greater variability in the reactivity against sera from Chagas patients than the peptides for CRA. The measured intensities plotted in Figure 2B suggested four potential epitopes from the sequences of the peptides presented in Panel C. Three of them (FRA-1, FRA-2 and FRA-3) consisted of sequences with only four amino acids (5**RRQL**8, 16**NAKE**19 and 26**SMNA**29). While their positions in the predicted model suggested that they were located at the surface of the proteins (Figure 2D), they were considered too small for use in the intended application as antigens for ELISA assays. However, the forth epitope (FRA-4) consisted of 14 amino acids (41**ADRAFLDQPERVP**54) that was predicted to form a random coil structure by the I-TASSER server. Its high level of reactivity suggested that it was a promising candidate for use as an antigen in ELISA for detecting antibodies associated with *T. cruzi* infections.

All potential candidates are listed in Table 1. A search was performed to compare these sequences against protein databases to predict their cross-reactivity in diagnostic assays. In Table 2, the proteins identified by the analysis are listed by name and accession number along with their source and percent identity. For CRA-1 and CRA-2, multiple proteins demonstrated identical sequences, but each also originated from *T. cruzi* suggesting the feasibility to use these epitopes as *T. cruzi* specific antigens. The same was not true for the epitope discovered in FRA. Although very reactive with sera from Chagasic patients, this sequence was not specific to *T. cruzi* based on the discovery of similar sequences contained in proteins from *L. braziliensis* and *L. infantum*.

**Table 1:** **Linear epitopes identified in CRA and FRA proteins of *T. cruzi *** **and their amino acid positions**

**Protein**	**Epitope sequence**	**Position**
**CRA**	**KQKAAEATK**	**CRA-1 (7–15)**
	**KQRAAEATK**	**CRA-2 (21–29)**
**FRA**	**RRQL**	**FRA-1 (5–8)**
	**NAKE**	**FRA-2 (16–19)**
	**SMNA**	**FRA-3 (26–29)**
	**ADRAFLDQKPERVP**	**FRA-4 (41–54)**

**Table 2:** **Specificity of the antigenic regions present in CRA and FRA sequences that were identified by the Spot-synthesis technique in comparison to similar sequences of those identified as antigenic for *T. cruzi *** **according to the Expasy data base (http://www.uniprot.org/)**

**Acession number**	**Protein name**	**Organism**	**Identity**
			** (%)**
***CRA-1 (KQKAAEATK)***			
**Q9NJH4**	**Antigen JL8**	***T. cruzi***	**100**
**Q4DV01**	**R27-2 protein, putative**	***T. cruzi***	**100**
**Q4DN44**	**Putative uncharacterized protein**	***T. cruzi***	**100**
**Q26947**	**R27-2 protein**	***T. cruzi***	**100**
**E7LGS7**	**Putative uncharacterized protein**	***T. cruzi***	**100**
**B9V414**	**Antigen**	***T. cruzi***	**100**
***CRA-2 (KQRAAEATK)***			
**Q4DV01**	**R27-2 protein, putative**	***T. cruzi***	**100**
**Q4DN44**	**Putative uncharacterized protein**	***T. cruzi***	**100**
**Q26947**	**R27-2 protein**	***T. cruzi***	**100**
**E7LGS7**	**Putative uncharacterized protein**	***T. cruzi***	**100**
**B9V414**	**Antigen**	***T. cruzi***	**100**
***FRA-4 (ADRAFLDQPERVP)***			
**Q04946**	**Cytoskeleton associated protein**	***T. cruzi***	**92**
**Q4CS87**	**Calpain cysteine peptidase, putative**	***T. cruzi***	**92**
**Q4CPQ6**	**Calpain cysteine peptidase, putative**	***T. cruzi***	**92**
**Q26898**	**Antigen DNA (tandem repeat sequence)**	***T. cruzi***	**92**
**E7LGE0**	**Calpain cysteine peptidase, putative**	***T. cruzi***	**92**
**A4HFX3**	**Putative calpain-like cysteine peptidase**	***L. braziliensis***	**90**
**E9AHC1**	**Putative cysteine peptidase**	***L. infantum***	**76**

### ELISA-peptide assay

The impetus for identifying the linear B-cell epitopes in CRA and FRA was to improve on their use as antigens for diagnostic ELISA assays. To verify their utility, peptides were synthesized from sequences derived from the potential CRA and FRA epitopes, the two major antigenic regions in CRA (CRA-1 and CRA-2) and the one in FRA (FRA-4), by solid phase synthesis using the F-moc strategy. Since both epitopes identified in CRA were 6-9-residues long, the potential for antigenic presentation in a diagnostic test was improved by synthesizing both with alanine residues as spacers (CRA-1 = AAKQKAAEAAAKQKAAEC; CRA-2 = AAKQRAAEAAAKQRAAEC). The FRA-4 epitope was already composed of 14 residues, so its sequence was not modified for synthesis (FRA-4 = ADRAFLDQKPERVPC). After synthesis, the peptides were conjugated with biotin at the C-terminus to allow easy attachment to ELISA plates through association with immobilized neutravidin.

**Figure 3:**
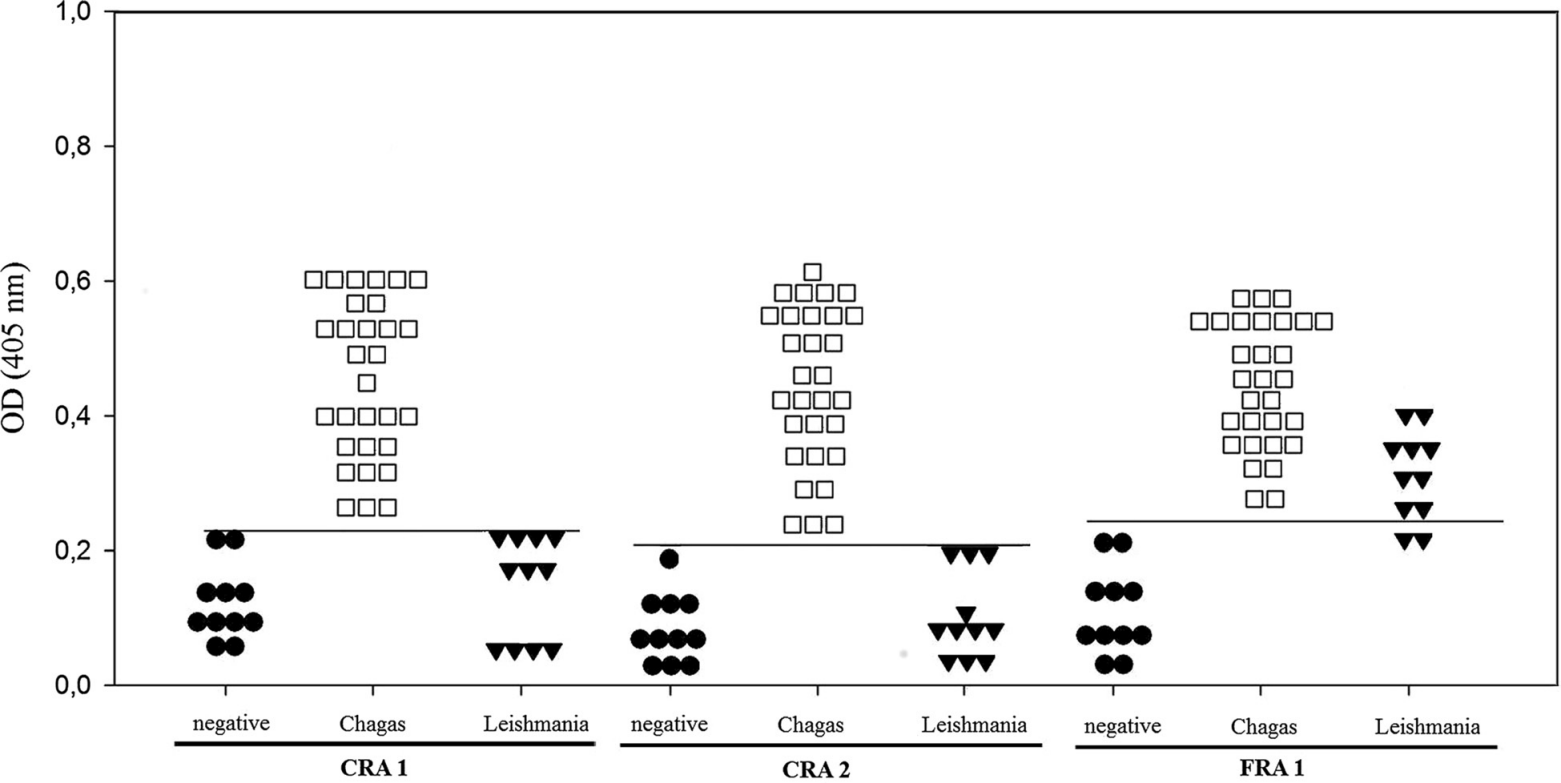
**Capacity of the (CRA-1, CRA-2 and FRA-4) synthetic peptides to discriminate between serums infected by *T. cruzi *and *Leishmania *in a ELISA.** Chagasic (n = 31) and Leishmaniasis (n = 14) patient sera, and healthy individual sera (n = 11) were diluted 1:50 and evaluated in triplicates against the peptide sequences CRA-1 (AAKQKAAEAAAKQKAAEAC-biot), CRA-2 (AAKQRAAEAAAKQRAAEC-biot) and FRA-4 (ADRAFLDQKPERVPC-biot). All tests were considered significant (p < 0,001) and the cut-off values of 0.226 for CRA-1, 0.199 for CRA-2 and 0.254 for FRA-1 were calculated. Each point of the graphic represents the media of two experiments.

To verify the diagnostic performance of the peptides, sera from thirty-one Chagasic patients (positive by indirect fluorescence), sera from fourteen Leishmaniasis patients and twelve healthy donors (negative) were tested on an ELISA-peptide based assay. The results are shown in Figure 3 and were considered significant (p < 0,001). Using the ROC curve analysis, the best values were associated with the CRA-1 and CRA-2 peptides and considered by a scale value as good test. Both of the CRA antigens demonstrated 100% sensitivity and specificity. The FRA antigen, however, presented a high cross reactivity with Leishmaniasis patient’s sera and by the ROC curve analysis represented a failed test by exhibiting a specificity of only 60% even with a sensitivity of 91.6%.

## Discussion

The development of improved assays for detecting *T. cruzi* infections, which can lead to Chagas disease, depends on the availability of specific, high-affinity antigens. In particular, useful antigens include those that are bound at equimolar levels to reactive antibodies present in serum and those that distinguish between antibodies derived against similar pathogens. The identification of antigens as strongly reactive linear epitopes and their characterization would permit their localization in protein models, definition of their biophysical properties and analysis of their binding properties with regards to antibody class, capacity, specificity and selectivity. Despite the determination of the RPs CRA and FRA as antigens for diagnostic purposes through various immunological studies [24,27,29,30,43], there are no published data about the number and composition of their epitopes at the amino acid level.

In our study, the mapping of linear epitope through parallel synthesis of overlapping peptides was chosen based on the volume of useful information provided and the high spatial resolution of the technique. Screening of the potential linear epitopes with sera from infected patients indicated the presence of two major antigenic and apparently highly specific epitopes in CRA when evaluated against the sera of Leishmanisis patients. The two epitopes had very similar sequences in comparison to each other. This result was similar to that of Pereira et al. [44], which demonstrated that the LamB fusion protein, a 14-residue peptide, was highly antigenic in mice and contained an immunodominant B cell epitope. Here, we were able to restrict the antigenic sequence to 9 residues, which is more easily obtained from solid-phase chemical synthesis. For FRA, the data demonstrated, for the first time that its recognition as an antigen was due to the presence of three small and one major antigenic region. Although the longest one consisted of 14 residues and was very reactive, its sequence also demonstrated a high similarity to *Leishmania sp.* proteins through a database search of Uniprot submissions. This high degree of similarity could explain its tendency to cross react with antibodies from patients infected with *Leishmania*.

After analyzing several antigen-antibody structural complexes, Rubinstein et al. [45] verified that B-cell epitopes are usually defined as regions with charged or polar residues and have hydrophilic characteristics with an undefined secondary structures, principally random coils and turns. The sequences mapped in CRA conformed to this characterization and were predicted to be very hydrophilic with a positive charge at neutral pH. In fact, the single amino acid substitution of arginine for lysine, defined between two epitopes in CRA, did not change any of the physical-chemical parameters. In addition, molecular modeling indicated that these epitopes were present in regions with undefined 3D structures as either a turn or a random coil. In contrast, the major FRA-4 epitope was calculated to have a slight negative charged, but also had a highly hydrophilic nature. The structural prediction indicated a random coil conformation. These results show that, in all three cases, the absence of a rigid 3-D organization may have contributed to the antigenicity of these sequences by increasing their exposure and availability to antibody binding.

Both CRA and FRA proteins have already been used as antigens in a variety of studies on the diagnosis of Chagas disease. Krieger et al. [25] published the first account to show that the sensitivity and specificity of ELISAs could approach 100% with a mixture of the recombinant forms of CRA and FRA as antigens. It also demonstrated that their diagnostic performance was improved when both proteins were used together rather than individually. A similar observation was made [28], using recombinant CRA and FRA on a microsphere-based serological test. However, when using a mixture of both recombinant CRA and FRA in ELISA for blood bank screening, Carvalho et al. [27] observed a decrease in the sensitivity of the test. This could be attributed to the observation that, when using whole proteins, a variety of epitopes are actually presented that could lead to some cross-reactions. An alternative to circumvent this issue is to develop a diagnostic test based on peptides as antigens, either individually, in mixtures or through multi-epitope structures. Some ELISA tests based on this approach have been tested for Chagas disease diagnosis. The results showed sensitivity in the range of 96.8 to 100% and specificity around 99% [4].

In this work, we were able to successfully use synthetic peptides in an ELISA-based diagnostic test for Chagas disease. The sequences of the peptides were defined by the reactivity of pooled sera from Chagas patients against a library of peptides designed to span coding region of CRA and FRA. To determine the best cut-off values for each test, a ROC curve analysis was chosen for the analysis because it permitted the researchers to fit test values and to achieve the best parameters according to defined objectives (for example, better sensitivity, better specificity or the best of both, simultaneously). With this approach, ELISA-based diagnostic tests that used either of CRA antigens achieved 100% sensibility and specificity, while FRA antigen showed a lower sensitivity of 91.5% and a specificity of 60%. This balance was pursued in order to overcome the fact that similar sequences could also be found in *Leishmania sp*. proteins (on a database search at Uniprot), thus avoiding cross-reactions on the test.

We expect that the localization and characterization of the association targets of anti-CRA/FRA antibodies will aid in further structural insights for development of more sensitive and specific assays for Chagas disease.

## Conclusion

The molecular characterization of epitopes on antigens exposes potential benefits, both from an applied and a basic research perspective. Native or recombinant antigens are powerful reagents for serological tests because they contain a large spectrum of epitopes that covert the multitude of variations presented by individual responses. However, there is always a balance between the inclusion of diagnostically relevant epitopes contained in the antigens versus cross-reactive epitopes. Our work focuses on identifying linear sequences within antigens recognized by antibodies in patient sera to precisely map each epitope and allows characterization of their performance as diagnostic tools. In this study, we have located epitopes in the CRA and FRA proteins from *T. cruzi*. The epitopes from CRA demonstrated high specificity, while the largest epitope from FRA displayed extensive cross-reactivity. Their utility in diagnostic kits was tested by a peptide-based ELISA and the CRA epitopes will be useful for refining diagnostic reagents with a design towards higher performance displaying greater sensitivity and specificity.

### Ethics statement

The study was approved by the FIOCRUZ (IFF 0071/10) study center ethics committee and conducted in accordance with Good Clinical Practice (GCP) and all applicable regulatory requirements including the Declaration of Helsinki. Written informed consent was obtained from all participants prior to study entry.

## Competing interests

The authors declare that they have no competing interests.

## Authors’ contributions

SGS, JBP and JRC designed the experiment. CGB and LPG performed lab work. SGS and DWPJr drafted the manuscript. All the authors read and approved the final manuscript.

## Pre-publication history

The pre-publication history for this paper can be accessed here:

http://www.biomedcentral.com/1471-2334/13/568/prepub
